# Unraveling differences in fecal microbiota stability in mammals: from high variable carnivores and consistently stable herbivores

**DOI:** 10.1186/s42523-021-00141-0

**Published:** 2021-11-04

**Authors:** Franziska Zoelzer, Anna Lena Burger, Paul Wilhelm Dierkes

**Affiliations:** Bioscience Education and Zoo Biology, Goethe University Frankfurt, Max-von-Laue-Str. 13, 60438 Frankfurt am Main, Germany

**Keywords:** 16S rRNA gene, Microbiota, Herbivores, Carnivores, Variability

## Abstract

**Background:**

Through the rapid development in DNA sequencing methods and tools, microbiome studies on a various number of species were performed during the last decade. This advance makes it possible to analyze hundreds of samples from different species at the same time in order to obtain a general overview of the microbiota. However, there is still uncertainty on the variability of the microbiota of different animal orders and on whether certain bacteria within a species are subject to greater fluctuations than others. This is largely due to the fact that the analysis in most extensive comparative studies is based on only a few samples per species or per study site. In our study, we aim to close this knowledge gap by analyzing multiple individual samples per species including two carnivore suborders Canoidea and Feloidea as well as the orders of herbivore Perissodactyla and Artiodactyla held in different zoos. To assess microbial diversity, 621 fecal samples from 31 species were characterized by sequencing the V3–V4 region of the 16S rRNA gene using Illumina MiSeq.

**Results:**

We found significant differences in the consistency of microbiota composition and in fecal microbial diversity between carnivore and herbivore species. Whereas the microbiota of Carnivora is highly variable and inconsistent within and between species, Perissodactyla and Ruminantia show fewer differences across species boundaries. Furthermore, low-abundance bacterial families show higher fluctuations in the fecal microbiota than high-abundance ones.

**Conclusions:**

Our data suggest that microbial diversity is significantly higher in herbivores than in carnivores, whereas the microbiota in carnivores, unlike in herbivores, varies widely even within species. This high variability has methodological implications and underlines the need to analyze a minimum amount of about 10 samples per species. In our study, we found considerable differences in the occurrence of different bacterial families when looking at just three and six samples. However, from a sample number of 10 onwards, these within-species fluctuations balanced out in most cases and led to constant and more reliable results.

**Supplementary Information:**

The online version contains supplementary material available at 10.1186/s42523-021-00141-0.

## Background

Due to intensive research in the field of microbiome science and further development of DNA sequencing, the tasks and importance of gastrointestinal microorganisms, especially the production of short-chain fatty acids (SCFA) serving the host organism as energy supply, are now well described [1–3]. In recent years, a lot of research has been conducted to analyze the composition and factors influencing the microbiome for various species using two different approaches. The first often-used study design focuses on a single species or on a specific taxonomic classification. Here, multiple samples per individual or species are analyzed, representing one or several time points. Especially farm animals e.g. cattle [4–6], pigs [7–9] or sheep [10, 11], have been largely analyzed due to their importance in agriculture. The microbiota of some wild species, especially highly endangered species such as black rhinos [12], koalas [13] and Tasmanian devils [14], has also been described in more detailed studies. The advantage of this study design is that the microbial composition and diversity of the species studied can be compiled in detail and comprehensively. Moreover, further factors influencing the microbial composition can also be determined in in-depth statistical analyses.

The second study design focuses on an overall comparison within or between groups of animals e.g. terrestrial [15–19] and marine mammals [20], amphibians [21] or birds [22]. In contrast to the former approach, studies involving numerous species are usually based on a smaller number of samples per species or collection site. A possible disadvantage of this approach could be non-representative results of these analyses due to the limited number of samples per species studied. Especially for studies on Carnivora, there are notable inconsistencies across different studies. For instance, two lion samples show a dominance of *Fusobacteria* and *Firmicutes* in one study [17], while three lion samples of another one lack of *Fusobacteria* and instead contain *Actinobacteria* [16]. A similar pattern occurs in studies on different tiger and fox subspecies. While about half of the samples in one study [17] consist of *Proteobacteria* and *Fusobacteria* respectively, another study found large differences for those microbial families [raref. The above-mentioned examples raise the question whether a minimum number of samples is needed to describe the microbiota of a carnivore species. In addition, the issue remains whether there are taxa that are more susceptible to microbial fluctuations, or whether this is due to specific bacterial species.

We aim to integrate the above-mentioned approaches by analyzing a comprehensive dataset of four major mammalian (sub-)orders (Canoidea, Feloidea, Perissodactyla and Ruminantia) to identify differences within or between those. As those animals each have a characteristic digestive system and rely on a different diet, they are well suited to test for variation in their microbial composition. The digestive tract of the Carnivora is short and—beside that of the Insectivora—also one of the least complex among mammals. It is characterized by a short intestine and colon, as well as a small cecum. Carnivora are among the hindgut fermenter, which have the highest microbial density in the appendix, colon and rectum [23, 24]. In general, individuals of this order show only slight adaptations to microbial fermentation, since they rely on easily digestible protein-rich nutrition and have lower glucose needs [25, 26]. Analyses of 16S rRNA gene have shown a low bacterial diversity in the stomach of carnivores, but that diversity increases steadily within the distal intestinal sections [27]. In contrast to carnivores, herbivores such as Perissodactyla and Ruminantia depend on microbial fermentation for cellulose and hemicellulose degradation. Perissodactyla, as hindgut fermenters, are characterized by a simple stomach similar to Carnivora, but in contrast have an enlarged large intestine to extend the retention time of food, as well as an enlarged cecum as the main place of microbial fermentation. Compared to monogastric animals, ruminants have a segmented stomach consisting of the rumen, reticulum, omasum and abomasum. In contrast to the Perissodactyla, ruminants are foregut fermenters, in which microbial fermentation mainly takes place in the rumen. While the small and large intestines are similar in size to the Perissodactyla, the cecum is reduced [28, 29].

In order to create such a widespread dataset, microbiome analyses of zoo-housed animals are suitable in different ways. First, it is necessary to know as many individual and environmental influencing factors as possible to create a representative dataset using multiple samples per species, individuals and collection sites. In this regard, zoos offer a nearly perfect environment because the general conditions such as nutrition, age and pedigree of the animals are well-known. Second, microbiome research is of great interest for the zoos to improve animal welfare. Finally, the microbiota influences a variety of physiological and behavioral processes and, accordingly, a healthy microbiota is correlated with an animal's fitness. Other aspects that are largely unclear so far include possible changes in the microbiome in specific situations such as animal transport, animal socialization or feed conversion. With a meaningful dataset, deviations from the species-specific references can be identified and potential treatments initiated.

## Results

In total, we analyzed 621 fecal samples of 31 zoo-housed carnivore and herbivore species, performing Illumina MiSeq paired-end sequencing of the V3–V4 region of the 16S rRNA gene. After quality filtering and read merging, the dataset consists of 12,651,811 sequences (2315–134,440 sequences per sample) with an average of 20,308 sequences per sample. Following the DADA2 pipeline in QIIME 2, we identified 21,058 different amplicon sequence variants (ASV), across all samples (2315 to 134,414 ASV’s per sample). The most common classified ASV represented 453,104 times in 329 samples and belongs to a *Clostridium perfringens* strain.

### Composition of fecal microbiota of major mammalian (sub-)orders

**Fig. 1:**
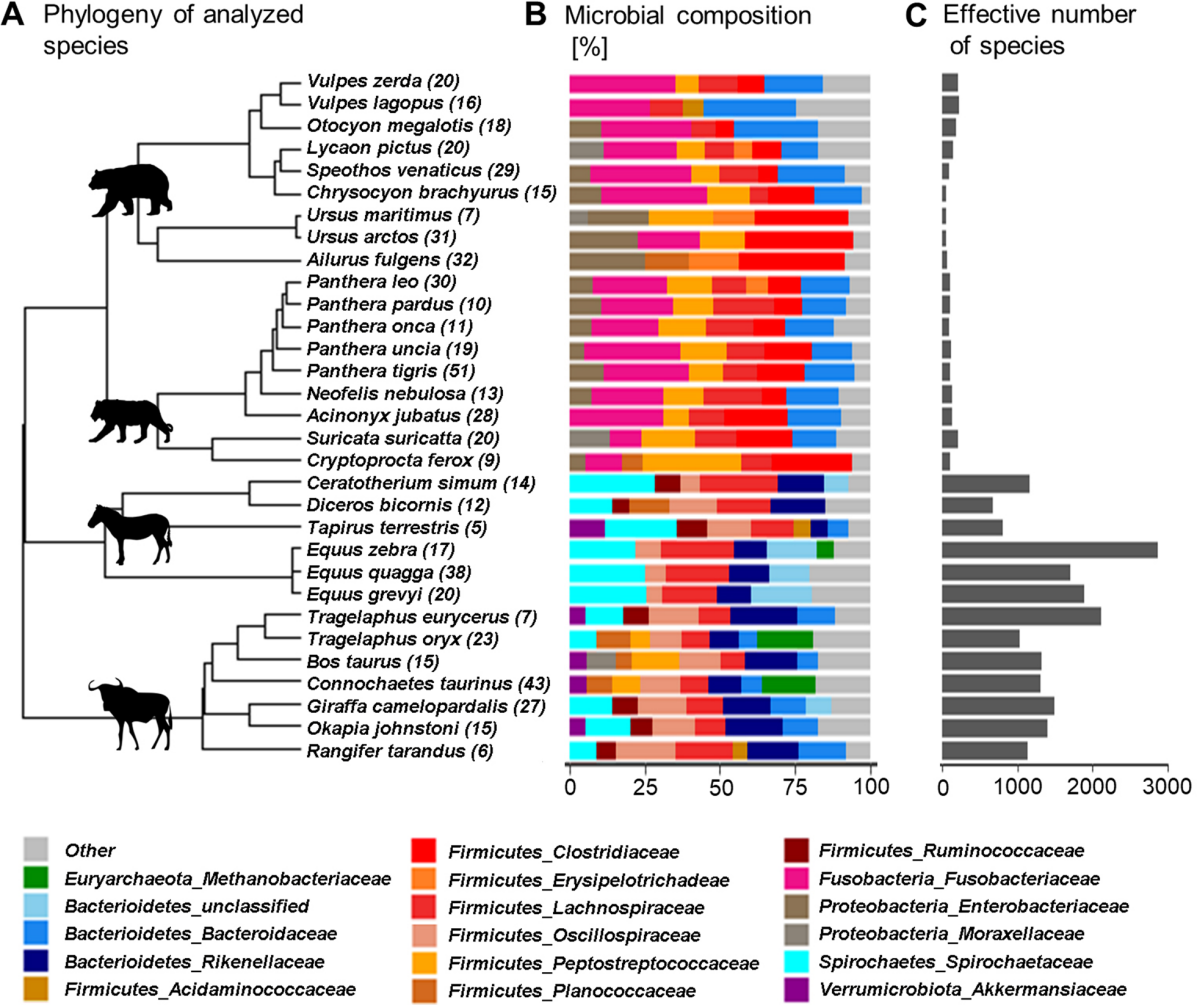
Variation in the fecal microbiota of mammals. **A** Phylogeny of the analyzed 31 species based on TimeTree database [30]. The total number of samples per species is shown in brackets. **B** Average composition of the fecal microbiota per species. Microbes that occur in less than 5% are summarized under “Other “. ANOSIM on the four groups: permutations = 999, distance = bray, R = 0.496, *p* < 0.001. **C** Average fecal diversity per species presented as number of effective species. Kruskal–Wallis on the four groups: *p* < 0.01, df = 3, Dunn Test with Bonferroni correction *p* < 0.001

We found significant differences between herbivores and carnivores in the microbial composition (ANOSIM statistic: R = 0.50, *p* < 0.001, number of permutations: 999, distance = "bray") as shown in Fig. 1B. As can be seen in this figure, the four major bacterial families across all herbivores are *Spirochaetaceae* (Average ± standard deviation: 15.3 ± 9.0%), *Lachnospiraceae* (15.3 ± 5.8%), *Rikenellaceae* (14.5 ± 4.4%) and *Oscillospiraceae* (12.4 ± 4.3%) (Additional file 2). Within the herbivores, *Spirochaetaceae* are more than twice as common in Perissodactyla (23.2 ± 4.4%) than in ruminants (8.5 ± 5.8%). While this family is equally distributed across perissodactylan species, within the ruminants it only occurs in larger proportions in giraffes (14.3%) and okapis (15.0%). In contrast, we found on average 20.2 ± 3.9% of *Lachnospiraceae* in Perissodactyla and only 11.1 ± 3.4% in ruminants, where larger proportions were observed in reindeer (18.9%). *Rikenellaceae*, the third most-common family in herbivorous species, constitutes on average to 16.1 ± 4.1% of the fecal microbiota of ruminants and to 12.6 ± 4.0% that of Perissodactyla. With respect to the *Oscillospiraceae*, we found notable differences between Ruminantia and Perissodactyla. While this family is equally abundant across nearly all ruminants (14.8 ± 2.7%), it only appears in tapirs (14.3%) and black rhinoceros (15.8%) in greater proportions of all Perissodactyla (9.6 ± 4.0%). Besides those four major families, we identified *Bacteroidaceae* in many ruminants (10.2 ± 3.4%) and an uncultured bacterium *p-251-o5* of the Bacteroidales order in Perissodactyla (9.7 ± 7.7%), especially in the grevy’s zebras (20.3%). Other bacterial families such as *Tanerellaceae*, *Erysipelotrichaceae*, *Clostridiaceae*, *Fusobacteriaceae* and *Enterobacteriaceae* constitute on average less than 5% of the microbiota across all herbivore species.

Furthermore, Fig. 1B illustrates that *Fusobacteriaceae* is the most dominant bacterial family in Carnivora species, occurring on average in 23.2 ± 7.1% of all Feloidea and in 22.38 ± 13.1% of all Canoidea. However, within the Canoidea, this family is low-abundant in red pandas and brown bears as it constitutes to less than 5% of both fecal microbiota. The distribution of *Clostridiaceae* (15.9 ± 10.1%), the second dominant family within the Carnivora, is on average similar for Feloidea (15.2 ± 5.8%) and Canoidea (16.6 ± 13.0%). *Clostridiaceae* form a large proportion of the microbiota, accounting for more than 30%, in both bears and red pandas. Those species also differ from other Canoidea with regard to *Bacteroidaceae*. Whereas this family is frequently found in most Carnivora (14.2 ± 8.9%), it is low-abundant (< 5%) in the red pandas, brown bears, polar bears and fossas. Additionally, we found on average 16.0 ± 6.5% *Peptostreptococcaceae* in Feloidea and only 8.5 ± 7.1% of this family in Canoidea, but the value calculated for Felidae is mostly influenced by its high abundance of 33.0% in fossas. Beside these major bacterial families, some others are largely represented in both bear species and red pandas. For example, we found that *Enterobacteriaceae* contribute on average 25.3% to the fecal microbial composition in red pandas, to 22.7% in polar bears and to 20.4% in brown bears. Furthermore, *Erysipelotrichaceae* are more dominant in brown bears (13.7%) and red pandas (16.5%) than in other Canoidea (4.0 ± 6.2%). With regard to the Felidae, *Lachnospiraceae* (14.0 ± 3.5%) are another dominant family, being equally distributed across all sampled felid species. Other bacterial families such as *Spirochaetaceae*, *Rikenellaceae* and *Oscillospiraceae*, which were dominant in herbivorous species, accounted for less than 5% of the carnivore microbiota.

### Microbial diversity within and between herbivores and carnivores

**Fig. 2:**
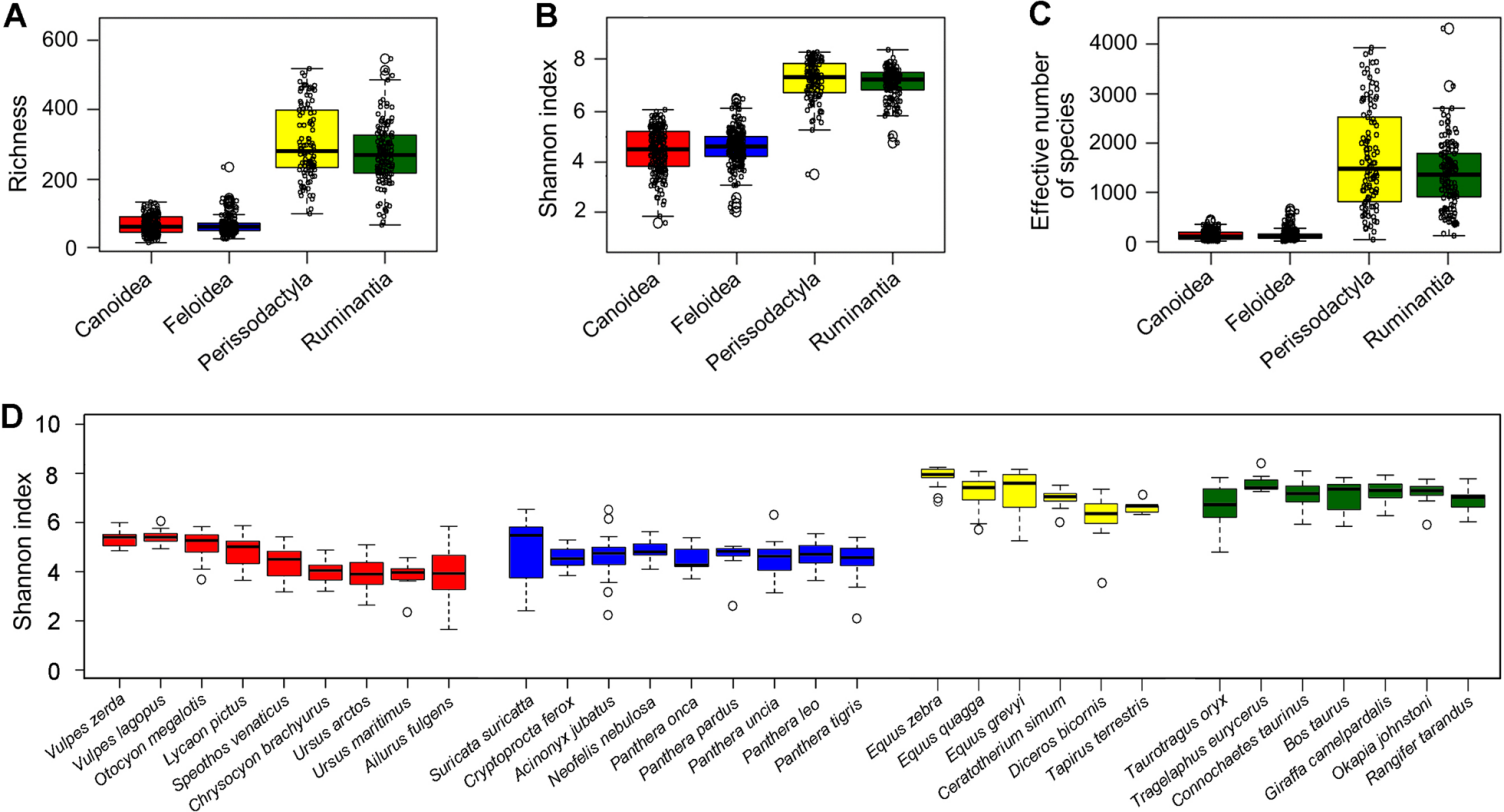
Alpha diversity of carnivores (Canoidea and Feloidea) and herbivores (Ruminantia and Perissodactyla) measured as species richness (**A**), Shannon index (**B**) and effective number of species (**C**). Part (**D**) shows the Shannon index for all analyzed species. Kruskal–Wallis *p* < 0.01, df = 3, Dunn Test with Bonferroni correction *p* < 0.001 for all diversity indices Statistical results for pairwise comparisons are presented in an Additional file 4)

The microbial diversity measured by effective number of species differs significantly between carnivores and herbivores as shown in Figs. 1C and Fig. 2C (Kruskal–Wallis: *p* < 0.001, df = 3, Dunn Test with Bonferroni correction *p* < 0.001), while there are no significant differences between Canoidea (90.0 ± 88.2) and Feloidea (101.1 ± 93.9) as well as between Perissodactyla (1475.9 ± 1030.5) and Ruminantia (1350.4 ± 673.3). Besides the ENS, those significant differences between carnivores and herbivores are further illustrated in the Shannon index and species richness (Fig. 2A, B). The median of the Shannon index is 4.5 ± 0.9 for Canoidea which is similar to Feloidea (4.6 ± 0.7) and significantly different (*p* < 0.01) to Perissodactyla (7.3 ± 0.8) and Ruminantia (7.2 ± 0.6). Furthermore, comparable results are obtained with the species richness (*p* < 0.01), which is more than four times higher in perissodactylan (279.0 ± 103.5) and ruminant species (268.5 ± 87.3) than in Canoidea (61.0 ± 27.0) and Feloidea (60.0 ± 25.5). Consequently, Carnivora species show a reduced microbial diversity over all measurements compared to Perissodactyla and Ruminantia species. Regarding the Shannon index across all species within a (sub-)order, further differences become visible (Fig. 2D). Within the Canoidea the greatest variation is found within the red pandas (3.9 ± 0.9). Additionally, the red panda samples show a significantly lower Shannon index compared to the Vulpini species represented by the fennec fox (5.4 ± 0.3, *p* < 0.001), arctic fox (5.4 ± 0.3, *p* < 0.001) and bat-eared fox (5.2 ± 0.6, *p* = 0.004). These three species generally show the highest alpha diversity within the Canoidea and differ significantly from the brown bear (3.9 ± 0.6, *p* < 0.001) and maned wolf samples (4.0 ± 0.4, *p* < 0.001). The Shannon index within the Feloidea species is very similar among species, and just the suricate samples show greater deviations (5.4 ± 1.2). Compared to some big cat species as the cheetah (4.7 ± 0.8), lion (4.7 ± 0.5), snow leopard (4.6 ± 0.7) or tiger (4.5 ± 0.5), the suricate samples show a significantly greater alpha diversity (*p* < 0.05). The zebras show the highest alpha diversity within the Perissodactyla, with the mountain zebra having a significant higher diversity (8.0 ± 0.4) compared to the plains zebra (7.4 ± 0.6, *p* < 0.05), tapir (6.7 ± 0.3, *p* < 0.05), black (6.5 ± 1.0, *p* < 0.001) and white rhino (7.1 ± 0.4, *p* < 0.001). Additionally, the highest variation was found within the grevy’s zebra (7.6 ± 0.9). The Shannon index within the analyzed ruminants is similar across all species. Only the elands (6.7 ± 0.8) show a significantly lower Shannon index compared to bongos (7.4 ± 0.4, *p* < 0.05) and wildebeests (7.1 ± 0.5, *p* < 0.05).

**Fig. 3:**
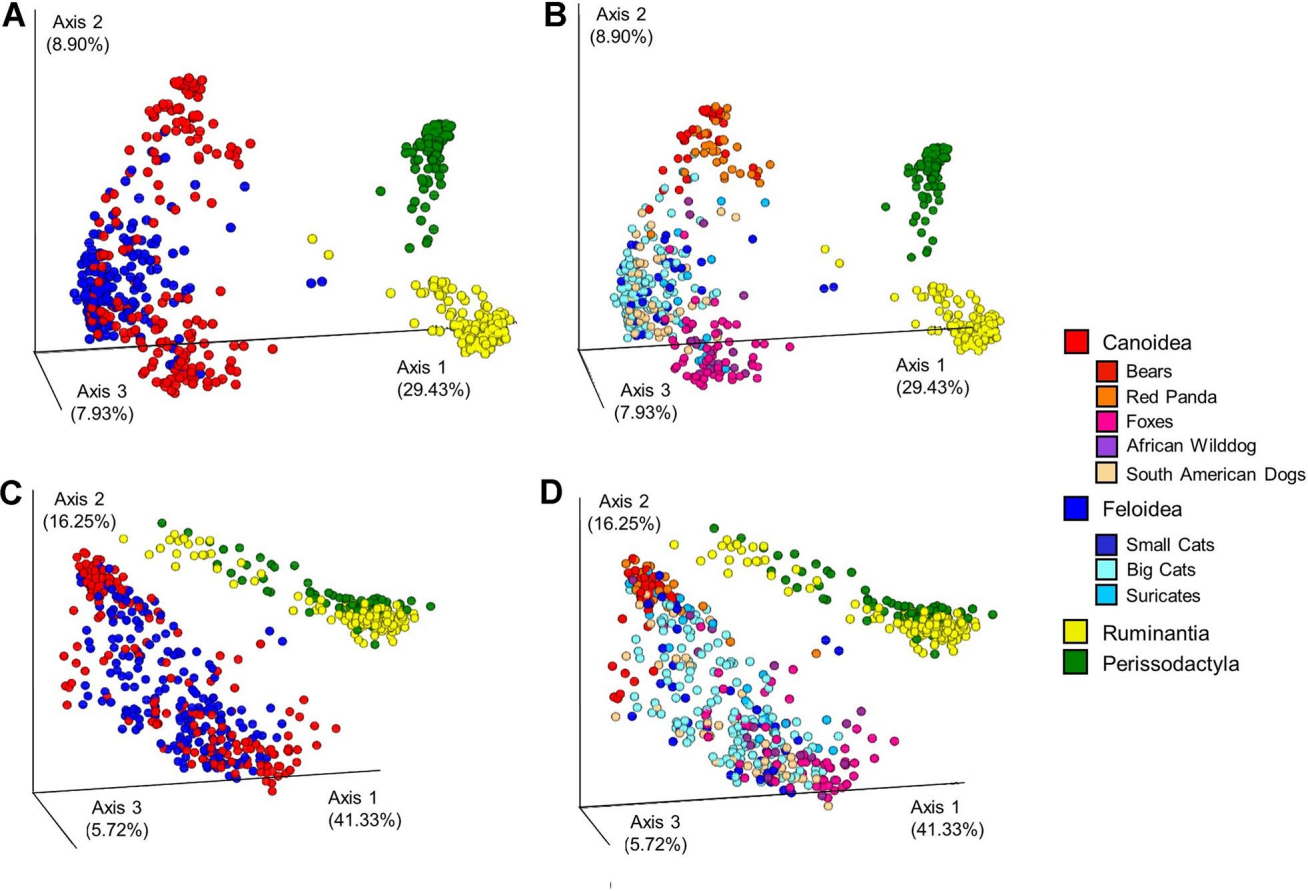
Principal Coordinates Analysis on the differences between carnivores and herbivores based on an unweighted and weighted UniFrac distance matrix. Differences in samples are shown based on the four analyzed groups (**A**, **C**) and on a more detailed division of carnivore groups (**B**, **D**) as shown in the figure legend. The proportion of data explained by this measurement is shown in brackets for each axis

Regarding the beta diversity, the principal coordinate analysis (PCoA) of the unweighted UniFrac distance matrix explains a total of 46.3% of data variability within the first three main axes (Fig. 3A, B), while the weighted UniFrac matrix explains a total of 63.3% of the data (Fig. 3C, D). Both plots show a clear separation between carnivores and herbivores, indicating a general difference in bacterial composition between these two groups. Homogeneity of dispersion is given within the four (sub-)orders (F = 0.670, *p* = 0.570, permutations = 999) and the ADONIS test shows significant differences in the fecal microbial composition between Canoidea, Feloidea, Ruminantia and Perissodactyla (R^2^ = 0.020, *p* < 0.001, permutations = 999). This is also confirmed by the PCoA of the unweighted UniFrac measurement (Fig. 3A, B). Similar to the weighted UniFrac, the homogeneity of dispersion is given for the animal (sub-)order (F = 0.670, *p* = 0.570, permutations = 999) and also for this metric, we found significant differences in the fecal microbial composition between the four(sub-)orders (R^2^ = 0.020, *p* < 0.001, permutations = 999).

Regarding the Perissodactyla and Ruminantia, both form clearer clusters in the unweighted UniFrac than in the weighted UniFrac measurement. This suggests that both, Perissodactyla and Ruminantia, can be distinguished by their general bacterial composition. Furthermore, in combination with the pattern observed in the weighted UniFrac plot, some differences within Perissodactyla and Ruminantia become visible which can be explained by the different abundance of some bacterial taxa. Thus, both herbivore groups consist of a similar microbiota that differs in the abundance of certain bacterial taxa. In contrast, there is no clear separation between Canoidea and Feloidea in either plot, indicating a differing bacterial composition within the Carnivora. In the unweighted UniFrac plot of the Carnivora (Fig. 3B), a slight pattern becomes visible. At the order-specific level, the Carnivora are divided into three clusters (Fig. 3B). The first cluster, closest to the Perissodactyla, consists of the polar and brown bear as well as the red panda samples. A little distant from these lies the center of the second cluster, made of the big and small cats as well as the South American Cerdocyonina represented by the bush dog and maned wolf samples. Finally, the third cluster, which is most distant from the herbivorous species, is composed of the Vulpini (fennec fox, arctic fox, bat-eared fox) and the African wild dog samples. Since these clusters are based on the unweighted UniFrac method, they can be distinguished from each other by a generally different bacterial composition. Since these clusters are less clear in the weighted UniFrac plot (Fig. 3D), these differences might be explained by the occurrence of low-abundant bacterial taxa, which are not visible when bacterial abundances are taken into account. Noticeably, four samples fall between the herbivores and carnivores, which belong to two elands from the same zoo and two cheetahs respectively. Since these animals were apparently healthy and did not differ in any other way from other sampled herd members, these outliers can at best be explained by a reduced read count (8204 and 7631 sequences for elands and 6,521 and 10,028 sequences). Regarding the two cheetahs, the general variability within the small cats is very high (Fig. 3B) and those samples might just underlie these deviations.

**Fig. 4:**
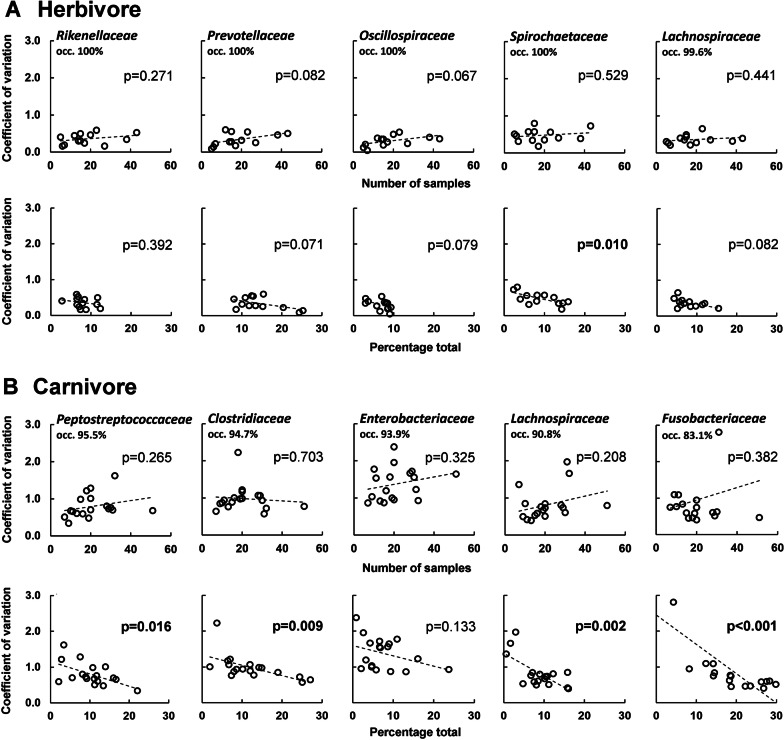
Coefficient of variation of different bacterial families for all herbivores (**A**) and carnivores (**B**) plotted against the number of samples (top row in each case) and against the total percentage of occurrence averaged per species (bottom row in each case). The tendency is indicated by a linear regression line and significant *p*-values are indicated in bold. The occurrence in the total sample is given for each bacterial family

**Fig. 5:**
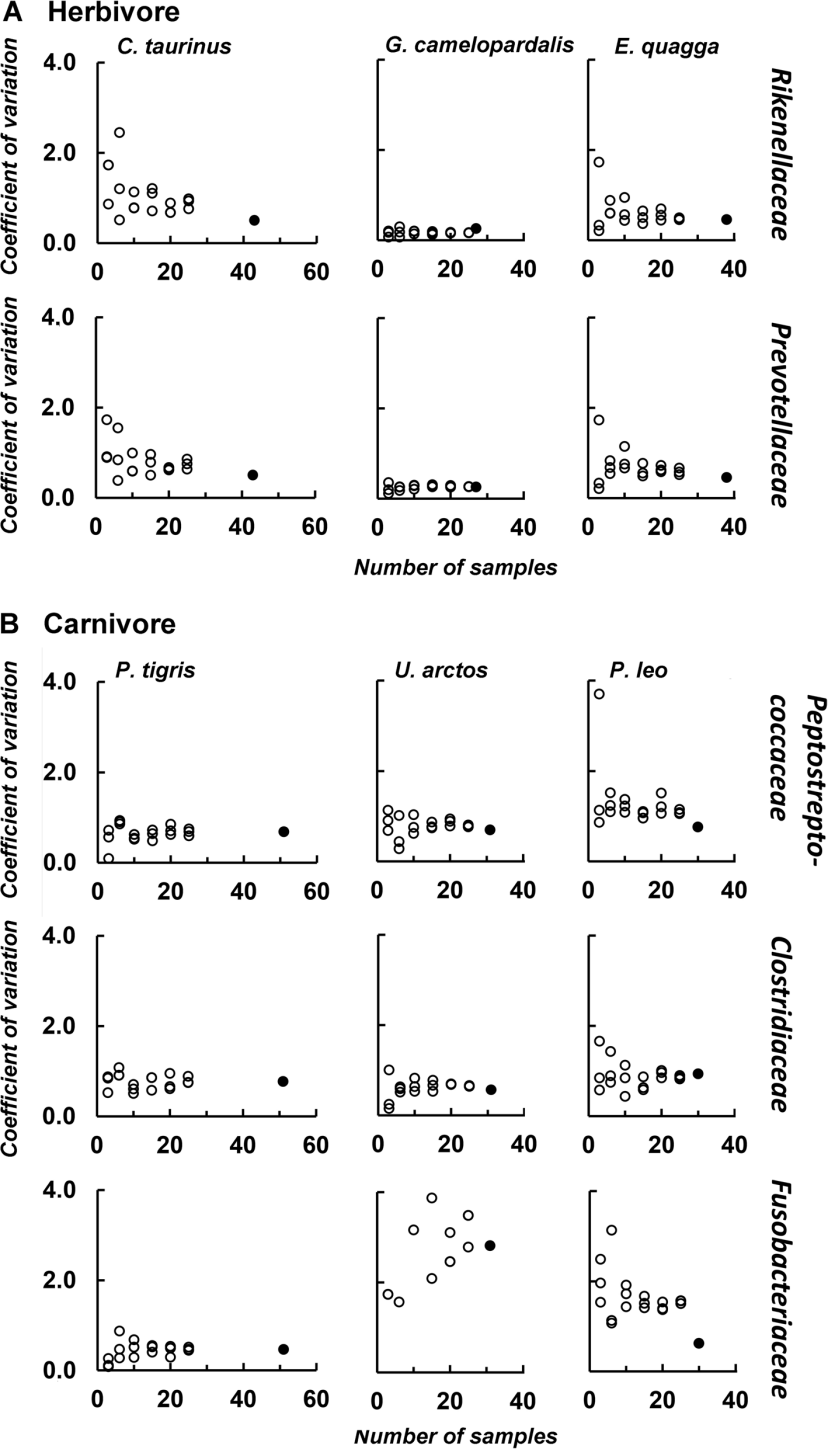
Coefficient of variation of different bacterial families for selected herbivorous (**A**) and carnivorous (**B**) species. Shown are randomized subsets (unfilled circles) for a different number of samples, as well as the entire data set (filled circles)

For a more detailed analysis of the variation within the Carnivora, we focused on fluctuations within the most common bacterial families, calculated as coefficient of variation (CV). The CV is defined as the ratio of standard deviation to the mean. Figure 4 shows the CV plotted against the number of samples and against the total percentage of occurrence of herbivores (4A) and carnivores (4B). These figures show three main results. First, the CV is in general lower for the illustrated bacterial families in carnivores compared to herbivores. Whereas the CV for the most dominant bacterial families within herbivores mostly not exceeds values of 1.0, the respective values within carnivores are about twice as high, e.g. for *Peptostreptococcaceae*, indicating higher variation within this bacterial family. Second, the relative variation (CV) of the low-abundant bacterial families (e.g., *Enterobacteriaceae*) is significantly greater on average per species than the variation of the high-abundant families (e.g., *Clostridiaceae* and *Fusobacteriaceae*), although the absolute variation of these bacterial families within the species studied is similar. Third, it is noticeable that the CV does not necessarily decrease with regard to a larger number of samples being analyzed, at least not when all herbivores or all carnivores are considered together. To examine whether this effect is possibly affected by species-specific differences, we created randomized subsets of bacterial abundance data for different sample numbers (n = 3, 6, 10, 15, 20, 25) with three replicates each, of three carnivore and herbivore species. For this purpose, we used bacterial families that occur in more than 7% of all herbivore or carnivore species, because low-abundant families seem to have a higher variability per se as shown before. Within all species, this results in a decreased coefficient of variation as the number of samples increases (Fig. 5). This clearly shows that when analyzing only a few samples per species (n = 3 or 6), there is generally greater variability in bacterial abundance data between samples than when using larger numbers of samples (n = 20 or 25). In addition, species-specific differences become visible. For example, giraffes show a constantly low variability in both bacterial families, even when only a few samples are considered. In contrast, wildebeests and plains zebras are more variable when only a few samples are taken into account and first stabilize at a sample number of 15 in both analyzed bacterial families. Within carnivores, the tiger samples show a constant CV for all bacterial families from a sample number of n = 10. Even if the variability within the lion samples is higher compared to the tiger ones, they also become stable from a sample number of 10 onwards. Besides species-specific differences, we also found differences in the variability between bacterial families in the brown bear. While the pattern for *Peptostreptococcaceae* and *Clostridiaceae* is the same as in tigers and lions, the high CV values of the *Fusobacteriaceae* is not noticeably declining with an increased sample size. Detailed results are shown in the Additional file 3.

To control for the zoo habitat as a possible influencing factor on the fecal microbiota, we performed a multinomial regression model on the microbial differential abundance data. The evaluation of the model setting ‘zoo’ against a null model obtained a Q^2^ value of 0.13. Compared to that, the model containing only ‘species’ as explanatory variable obtained a Q^2^ value of 0.33. A combined model (‘zoo’ + ‘species’) results in a slightly higher Q^2^ value of 0.43. In order to distinguish the obtained zoo effect more precisely from the effect of the variable ‘species’, we compared the model including ‘zoo’ as a variable against a baseline model containing ‘species’ as a variable. This results in a negative Q^2^ score, illustrating that the variable ‘zoo’ does not improve the model when ‘species’ is set as a baseline.

### Microbial indicators for herbivore and carnivore animals

Indicator families were analyzed for each of the four (sub-)orders and each possible combination using the IndVal.g function. We identified a total of 276 indicator families, most of them for herbivores, especially for Perissodactyla (Table 1). With 18 indicator families, Canoidea and Feloidea share less indicators than Perissodactyla and Ruminantia and only minor proportions of indicator families were found in combinations of herbivore and carnivore species. The complete results are presented in the Additional file 5.

**Table 1:** Microbial indicators for different animal (sub-)orders and their combination

(sub-)order	Number of indicator species
Canoidea	10
Feloidea	6
Perissodactyla	43
Ruminantia	16
Canoidea + Feloidea	18
Perissodactyla + Ruminantia	42
Canoidea + Perissodactyla	1
Canoidea + Ruminantia	3
Feloidea + Perissodactyla	2
Canoidea + Feloidea + Perissodactyla	3
Canoidea + Feloidea + Ruminantia	6
Canoidea + Perissodactyla + Ruminantia	4
Feloidea + Perissodactyla + Ruminantia	2

Indicators were assigned at microbial family level

Almost all predicted indicator families show high A values, meaning that this indicator only occurs in the tested (sub-)order, but is not necessarily spread across all of its members. In contrast, the B values, showing the distribution of an indicator across all taxa, are much more variable. Indicator families restricted to Canoidea are *Gemellaceae* (A = 1.00, B = 0.03) and *Xiphinematobacteraceae* (A = 1.00, B = 0.02), but they do not occur in all the samples. Regarding the Feloidea, no exclusive indicators were found. However, *Coriobacteriaceae* (A = 0.88, B = 0.88) are strongly related to this suborder and distributed among nearly all members. In general, all indicator families associated to the Carnivora show low B values, which might be a further indication of greater diversity within the two suborders as seen in the PCoA analysis. However, this view changes when one considers the indicator families that occur in both the Feloidea and the Canoidea. In particular, *Enterobacteriaceae* (A = 0.98, B = 0.94), *Clostridiaceae* (A = 0.96, B = 0.95) and *Fusobacteriaceae* (A = 0.99, B = 0.83) occur in almost all Carnivora species and appear to be clear indicator families for those in general. Additionally, these families are also the most dominant ones in the Carnivora fecal microbiota composition (Fig. 1b).

In contrast, more indicator families were found in Perissodactyla and Ruminantia. *Fibrobacteraceae* (A = 0.81, B = 0.97), *Synergistaceae* (A = 1.00, B = 0.75), *Defluviitaleaceae* (A = 0.88, B = 0.80) and *Methanocorpusculaceae* (A = 0.79, B = 0.88) occur almost exclusively in Perissodactyla and are present in almost all species. For ruminant species, one of the most prominent indicators are *Barnesiellaceae* (A = 0.89, B = 0.72) and *Atopobiaceae* (A = 0.73, B = 0.46), which occur in many members of this suborder. Looking at the combined indicators of Perissodactyla and ruminants, many microbial families are found almost exclusively in those two (sub-)orders and are present in all taxa. Again, those indicator families are among the most dominant ones in the taxonomy plot (Fig. 1b) i.e. *Spirochaetaceae* (A = 0.99, B = 1.00), *Rikenellaceae* (A = 0.96, B = 0.99) and *Oscillospiraceae* (A = 0.87, B = 0.90).

## Discussion

The aim of this work was to conduct a study on the variability of the microbiota of zoo-housed carnivore and herbivore species, with a focus on the four (sub-)orders Canoidea, Feloidea, Perissodactyla and Ruminantia. In contrast to previous studies using just a few samples per species, we analyzed multiple samples per species and compared the microbiota of species from different locations. Our study results in two main findings. Firstly, we found significant differences in the microbiota composition of carnivorous and herbivorous species, as well as a significant higher alpha diversity in herbivores. Secondly, we found closer similarities and less variability in the fecal microbiota of Perissodactyla and Ruminantia compared to higher deviations in Carnivora, which has some important methodological implications as discussed below.

### Differences in the microbiota composition between carnivores and herbivores

We found significant differences in the fecal microbial composition between herbivore and carnivore species. The most dominant bacterial families found in herbivore species are *Spirochaetaceae*, *Lachnospiraceae*, *Rikenellaceae* and *Oscillospiraceae*. The first two mentioned occur more frequently in Perissodactyla, whereas the latter two appear on average more often in ruminants. Those results are in line with the in-depth study on African herbivores [19], who also found *Oscillospiraceae* as the most dominant family in ruminants such as giraffes, cattle or hartebeests. Nevertheless, our study showed greater proportions of *Rikenellaceae* in ruminants. Both, *Oscillospiraceae* and *Rikenellaceae*, have recently been characterized as herbivore specific bacteria in a covariance network analysis [18], with *Oscillospiraceae* being a major player in cellulose degradation and therefore being related to a herbivore and fiber-rich diet [31]. Another link to the study on African herbivores [19] is the appearance of *Spirochaetaceae*, especially in zebras, as representatives of Perissodactyla. Similar to *Oscillospiraceae*, this family is responsible for fiber digestion and therefore essential for the herbivore digestive system [32, 33]. Besides *Spirochaetaceae*, we found *Lachnospiraceae* as another main family in Perissodactyla. This family has been detected in the human intestine as well as in the rumen and digestive system of different mammals [34, 35]. Bacteria belonging to this family such as *Roseburia* or *Lachnospira* are involved in the production of SCFAs by hydrolyzing sugars (e.g. starch) and were found to be associated with the consumption of plant protein and fiber [36, 37]. Additionally, the abundance of *Lachnospiraceae* can decrease with regard to a high-protein diet, indicating a minor role in protein metabolism [38]. Those major bacterial families found in herbivorous animals are mainly capable of carbohydrate digestion like starch or maltose, allowing the host to gain enough energy from the plant-based diet.

In contrast, the main bacterial families found in Carnivora are *Fusobacteriaceae*, *Clostridiaceae*, *Bacteroidaceae* and *Peptostreptococcaceae*. *Fusobacteriaceae* are often linked to a high-fat and protein-based diet and were observed in different carnivores, with *Fusobacterium* previously being classified as a carnivore specific bacterium [18, 39]. This bacterial family is able to produce SCFAs using carbohydrates or amino acids [40] and it has been shown that *Fusobacteriaceae* are more common in carnivorous Carnivora than in omnivorous or herbivorous Carnivora [41], which is consistent with our study. Both, *Clostridiaceae* and *Bacteroidaceae*, being dominant in carnivore families in our study have already been detected in the gastrointestinal microbiota of different predators [18, 39, 42, 43]. While *Clostridiaceae* appear to be important for protein metabolism, *Bacteroidaceae* occur in combination with a fiber-rich diet and are not affected by protein intake [44–46]. In summary, our results show the highest proportion of *Bacteroidaceae* in bat-eared foxes as well as the highest proportion of *Clostridiaceae* in polar bears, which partly matches this theory. However, we could not find major differences for these two bacterial families.

Beside significant differences in the microbial taxonomic assignment between carnivorous and herbivorous mammals, we also found a significantly higher microbial alpha diversity in Ruminantia and Perissodactyla compared to Carnivora. This might be due to the more complex digestive system of herbivorous species and their dependence on microbes to break down cellulose. This relationship has been shown previously for several species [35, 39, 41, 47, 48]. Furthermore, herbivorous mammals are known to rely on microbial metabolic pathways to a greater extent than carnivores [18].

In addition to confirming previous studies on the carnivore microbiota, we have also found some species that deviate from previous assumptions, namely both bear species, the red panda and the fossa. Contrary to the other Carnivora, *Fusobacteriaceae* only occur in minor proportions within red pandas and brown bears, but *Erysipelochtrichaceae* are enriched in these animals. Furthermore, both bear species and the red pandas consist of major proportions of *Enterobacteriaceae* but only of minor proportions of *Bacteroidaceae*—similar to the fossa. Within the PCoA plot of beta diversity (Fig. 3B), the fossa samples lie within those of other felids, whereas the two bear species as well as the red pandas form a separate cluster apart from the Feloidea and the Canidae. The most influencing factors for fecal microbiota composition are described to be diet and phylogeny [17, 32, 49]. Because the omnivorous diet of the analyzed bears was similar to that of the other Canoidea as e.g. the Vulpini species which form an own cluster, and even the red pandas were fed an omnivore diet in half of the analyzed zoos, it is unlikely, that this separation is mainly influenced by diet. Another factor influencing the microbial composition is the host phylogeny. Bears, red pandas and fossa all evolved separated from other members of the respective suborder. The fossa as a Malagasy carnivore evolved distinct from other Felidae as a sister clade to the Herpestidae about 18–24 Mya ago [50, 51]. Regarding the Caniformia, the Arctoidea clade split in a rapid radiation about 43 Mya in three superfamilies Ursoidea, Pinnipedia and Musteloidea. Within these, the Ursidae evolved about 18 Mya ago, whereas the Ailuridae evolved about 33 MYA ago as a sister clade to Mephitidae, Procyonidae and Mustelidae [52–55]. In recent years, the theory of co-evolution between host and microbes arose and continues to be proven. It states that bacterial symbionts adapt to e.g. dietary changes of the host and the host in turn adapts to the changed microbiota or that allopatric speciation of the host might even lead to co-phylogenetic patterns between microbes and host [16, 17, 56–58]. Although this was not analyzed in this study, our results may suggest a co-evolution between gut microbes and host phylogeny in different mammalian (sub-)orders. Furthermore, the results indicate that there are clear differences between herbivore and carnivore species but that there are several deviations from previously published gut microbiota.

## Close similarity in the fecal microbiota of herbivores and great diversity within the carnivores

Beside significant differences between herbivore and carnivore species, our results reveal a closer similarity in the fecal microbiota of Perissodactyla and Ruminantia compared to higher deviations in Carnivora. Although there are several studies that describe either a distinct clustering of herbivores and carnivores due to differences in diet or phylogeny or a clustering of herbivorous carnivores to other Carnivora [16, 39, 41, 47, 49], none of them has yet referred to the variability of the microbiota within these taxa. A first indication of greater uniformity within Perissodactyla and Ruminantia is the larger variety of indicator species than for Carnivora, which can be explained with an overall higher alpha diversity as well as a closer similarity of the fecal microbes in herbivores. Furthermore, the Carnivora indicators are not distributed equally across all species, indicating a greater intra- and interspecies variation within this order. These differences are further illustrated in Figs. 4 and 5 showing the coefficient of variation within herbivores and carnivores. Here, the coefficient of variation is much higher in low-abundant microbial families compared to high-abundant families. One explanatory approach for the higher deviation within the Carnivora is the diet. While the analyzed herbivores are mostly fed on hay, alfalfa or grass throughout the year, the diet and its composition is more variable in carnivores. Especially omnivorous Carnivora such as most Canoidea are fed on a variety of food sources as fresh and kibble meat, fruits, vegetables or insects. But even hypercarnivore species undergo daily changes in meat origin or preparation (e.g. whole-body or sheer meat). For canids and felids, it is shown that the fecal microbiota is greatly altered by diet and dietary changes. Especially changes in the proportion of carbohydrates and protein influence the necessary gut bacteria, i.e. *Prevotella* or *Fusobacteria* respectively [42, 43, 59–61].

These differences in the microbial variability of carnivorous fecal samples also have important methodological implications. It is therefore necessary to adapt the number of samples being analyzed to the species to be studied in order to obtain meaningful results. Herbivores are very similar in terms of their microbial composition. In ruminants, *Oscillospiraceae*, *Lachnospiraceae,* and *Rikenellaceae* appear to dominate as the major bacterial families [16, 19] and this is evident in studies using different sample sizes. For example, the core results of a study on five giraffe samples are consistent to a similar study on more than 50 giraffe samples and the same pattern can be seen in regard to studies on elands [19, 47, 62] or zebras representing Perissodactyla [16, 19]. These results are in line with the low CV that we found in herbivores. Nevertheless, we found species-specific differences for the major bacterial families within herbivores as well. Giraffes show very low variability in *Rikenellaceae* and *Prevotellaceae,* so these differences should be visible even in very few samples analyzed. In contrast, wildebeest samples are highly variable for those two families, resulting in the need to analyze at least 15 samples to control for these variations.

The Carnivora microbiota in general is much more variable, which is expressed in a higher CV compared to that of Perissodactyla and Ruminantia. Especially within this order, it is therefore important to analyze a reliable number of samples in order to characterize the microbiota. This is also illustrated by the fact that previous studies on carnivores yield significantly different results on the composition of the fecal microbiota. For example, studies using just two or three fecal fox, polar bear or bush dog samples [16, 47] found great differences in the proportion of *Prevotellaceae* and *Fusobacteriaceae*. The same pattern was observed for Feloidea, in studies on just a few cheetah and lion samples which could only detect minor proportions of *Fusobacteria*, whereas a study using more than 60 animals reported about 20% *Fusobacteria* in cheetahs [16, 63–66]. In this study, we found *Fusobacteriaceae* across all Carnivora species in highly different proportions. Within the brown bear samples, this family is present on average in 4.3%, which explains the high coefficient of variation even when using a high amount of samples. But also within the lion and tiger samples, in which the proportion of *Fusobacteriaceae* with an average of 18.3% and 23.5% is considerably higher, the CV for this family only becomes constant with 10 samples being analyzed (Fig. 5). This strengthens our finding that low-abundant bacterial families are more variable in the fecal microbiota of mammals, and the necessity of analyzing multiple samples to reduce uncertainties that can occur with small sample numbers (n = 3 or 6).

Considering the highly variable microbiota of Canoidea and Feloidea and the more constant microbiota of Ruminantia and Perissodactyla, it is important to select an appropriate number of samples for further analysis. Depending on the methodological approach, it should be noted that low-abundant bacterial families are often subject to greater fluctuations than high-abundant ones, and that there seem to be species-specific differences in microbiota variability within these animal (sub-)orders.

### External influencing factors on the microbiota of zoo-housed animals

An often mentioned criticism on the microbiome analysis of zoo animals is the fact that captivity might lead to a reduced microbial alpha diversity in some species [67, 68]. Reasons for this may include a different dietary composition, the use of additives and medicines, or the artificial enclosure design. To address this point of criticism, we have compared some of our data with the methodologically comparable study by McKenzie et al. [69]. They stated, that not all mammalian families are affected equally by a loss of microbial diversity as an effect of captivity. For example, the authors found a significant decrease in the Shannon index in canids. In our dataset, canids of the same species show a Shannon index which is higher than that of their captive samples and which is even more similar to the wild samples. Furthermore, the authors mentioned *Bovidae* and *Giraffidae* not to be impacted by captivity as they obtained comparable Shannon values in the wild and in captivity. Here too, our results are comparable with the diversity measurements of their wild samples. Another interesting finding of their study is that the alpha diversity of captive *Rhinocerotidae* is even increased, we calculated a Shannon index that is very similar to those enlarged value for captive rhinos. Even though the alpha diversity is only one component in the analysis of the fecal microbiota, and a comprehensive comparison would of course need to include the sample’s taxonomic composition as well as beta diversity, these results provide first indications for a better understanding of the microbiota diversity of zoo animals.

Nonetheless, our primary goal is to generate a dataset that contains numerous mammalian species, with a defined number of samples per species from different locations (zoos) to get an overall view of species-specific deviations in the fecal microbiota. Even if some species are subject to the captivity effect of reduced microbial diversity, all the samples are equally affected by this and therefore the results themselves are not biased. Rather, the respective zoo could be an external influencing factor on the fecal microbiota and to control for this effect, we conducted a multinomial regression. Regarding the whole dataset of microbial abundance data, the species-specific effect outweighs the effect of the housing location (zoo). Nevertheless, the respective zoo has slight influence on the fecal microbiota which can be caused by for example different feeding regimes, co-habitation and interaction of different species or the enclosure equipment. Furthermore, this zoo-specific effect differs between species and ranges from zero effects (e.g. Cheetah, Red panda) to greater effects in wildebeests or suricates. However, as we only focus on zoo-housed animals and our main focus in this study is not to compare those samples to samples from free-ranging animals, the housing location as influencing factor should balance out across all zoos. Nevertheless, we are aware that the microbiota of wild animals may differ from our results, and our findings clearly relate to captive animals. For them, however, they provide a comprehensive database on which further research can be conducted.

## Conclusions

To the best of our knowledge, this is the first study focusing on the microbiota variability of a wide range of carnivore and herbivore mammals by analyzing multiple samples per species in different locations. Our results support already existing theories such as a greater alpha diversity in herbivores or the general description of major bacterial families in Perissodactyla and Ruminantia species. Additionally, we found some species as the brown and polar bear, red panda or fossa that deviate from other members of their diet group. Phylogeny and host-microbe co-evolution may have a greater effect on fecal microbial composition here. In addition, we show that the microbiota of ruminants and Perissodactyla is more similar within the respective (sub-)order than within Carnivora. This results in a lower minimum number of samples that need to be analyzed to decipher the total fecal microbial diversity. For most of the bacterial families and animal species studied, our results show larger deviations when only a few samples (n = 3 or 6) are considered. In general, these deviations become smaller when 10 samples or more are considered and should thus be sufficient to provide a good insight into the fecal microbiota.

For further research, it will be interesting to investigate whether the greater variability of the Carnivora microbiota also applies in short-term time series analyses of a few days and which bacterial families remain constant or contribute to daily fluctuations in the fecal microbial composition.

## Methods

### Sample collection

Between April 2018 and August 2020, 621 samples were taken from 31 carnivore and herbivore species in a total of 20 German zoos (see Additional file 1). Non-invasive sampling was mostly performed during the daily cleaning routines of the enclosures in cooperation with the keepers. The samples were collected across four animal (sub-)orders, including Canoidea and Feloidea as representatives of the Carnivora, as well as Perissodactyla and Artiodactyla (only Ruminantia) as herbivores. For each species, a minimum of five samples across at least three different zoos was collected (except for *Vulpes lagopus*, *Equus zebra* and *Panthera onca*) When individual differentiation was not possible, fresh samples were collected from different locations in the enclosure to increase the likelihood that the samples are derived from different individuals. Only fresh fecal samples of different individuals were collected in previously disinfected 50 mL centrifuge tubes using sterile inoculation loops. In the next step, a subsample was taken from the center of the feces and transferred to a sterile 2 mL cryotube, which was then immediately stored in liquid nitrogen. All applicable international, national, and/or institutional guidelines for the care and use of animals were followed by the zoos. For further processing, the samples were delivered to StarSEQ GmbH in Mainz, Germany. Here, the samples were preprocessed with the Precellys® Evolution Homogenizer (Bertin Instruments, Rockville, USA) and DNA extraction was performed using the QIAamp® PowerFecal DNA Kit (Qiagen, Hilden, Germany). The DNA concentration in all extracts was measured using a NanoDrop spectrophotometer (Thermofisher, Massachusetts, USA).

### 16S rRNA gene sequencing and data processing

PCR amplicons for the V3–V4 region of the 16S rRNA gene were generated with primer pair 341F and 806R. Pooled amplicons were sequenced with the Illumina MiSeq 2 × 250 v3 kit for 600 cycles at StarSEQ GmbH. To control for sequencing quality, a 25% PhiX control library was added to the run. Samples were processed following the QIIME 2 [70] pipeline. After demultiplexing, DADA2 [71] was used to call amplicon sequence variants (ASVs) which reflect the biological sequence without clustering similar sequences on a given threshold. A phylogenetic tree was inferred for all sequences based on a sequence alignment generated by MAFFT and low-abundant ASV’s that occurred less than 10 times in the total data set as well as chloroplast and mitochondrial sequences were removed from the dataset. The taxonomic assignment of ASVs was performed using a pre-trained naive Bayes classifier [72] based on SILVA 138 full-length sequences [73]. The following statistics were performed in R version 3.6.3 [74] using the packages vegan [75] and FSA [76]. To test for differences in the taxonomic composition between the four mammalian (sub-)orders, ANOSIM test was performed on dissimilarity matrices with Bray–Curtis distances. Alpha diversity was determined by Shannon index, the effective number of species (ENS) [77, 78] and richness which were calculated using QIIME2 after rarefying the number of reads per sample to a total of 2,300 reads. Afterwards, differences between groups were tested using the Kruskal–Wallis test, followed by a post-hoc Dunn Test with Bonferroni correction in R. Beta diversity was also calculated in Qiime2 core-metrics on the rarefied ASV table using unweighted and weighted UniFrac distances. Subsequently, a test for homogeneity of dispersion and the Adonis test for differences between groups was performed on the four (sub-)orders as well as on diet type (herbivore, carnivore). To calculate differences in the occurrence of bacterial families within carnivores and herbivores, the coefficient of variation (CV) was calculated for the respective major bacterial families. The coefficient of variation is a measure of relative variability of sample data and is calculated as the ratio of the standard deviation to the mean. An advantage of this measurement is that it is unitless and independent of the data scaling, which makes it particularly well suited to describe the dispersion of a parameter (here the abundances of individual bacteria families). For further analyses, subsets of the taxonomic assignment of the wildebeest, giraffe, plains zebra, tiger, brown bear and lion were created. Samples of the respective species were randomly drawn until a total sample number of 3,6, 10, 15 and 25 was reached. In addition, three replicates were created for each of these subsets.

For the most-abundant bacterial families, the CV was calculated on those replicates. To control for zoo as a possible influencing factor on the fecal microbiota, we performed a multinomial regression model on differential abundances using Songbird [79]. On the one hand we applied the model on the whole dataset setting ‘zoo’ and ‘species’ as explanatory variables and evaluated this against a null model. On the other hand, the same regression was performed on a species-specific subset of microbial abundance data set as dependent variable and ‘zoo’ as explanatory variable. Furthermore, indicator species for each (sub-)order were identified using the indicspecies R package [80]. The IndVal value calculates the associations between species and sites, followed by a permutation significance test (n = 999, α = 0.05). Indicators were assigned at microbial family level. To create an approximate host phylogeny, the TimeTree database was used on the involved species names.

## Supplementary Information

**Additional file 1**. Sample metadata. Metadata of analyzed fecal samples according to MIMARKS host-associated package (version 5.0).

**Additional file 2**. Taxonomic assignment. Taxonomic assignment according to SILVA database (version 1.38) provided for each species. Furthermore, the microbial composition for all samples of one representative species per analyzed (sub-)order is shown. Those representatives are the brown bear (Canoidea), lion (Feloidea), wildebeest (Ruminantia) and plains zebra (Perissodactyla).

**Additional file 3**. Randomized datasets on the most abundant bacterial families in selected herbivore and carnivore species. For each species (plains zebra, giraffe, wildebeest, brown bear, lion, tiger) random sample ID’s were chosen for n = 3, 6, 10, 15, 20 and 25 samples to calculate the coefficient of variation for the bacterial families.

**Additional file 4**. Statistical tests on alpha diversity between herbivores and carnivores. Results of the significance tests for richness, Shannon index and effective number of species tested on Canoidea, Feloidea, Perissodactyla and Ruminantia. Non-parametric Kruskal-Wallis rank sum test and post-hoc Dunn test were used to analyze for differences between those groups.

**Additional file 5**. Indicator species results. The data describes the results of the indicator species analysis. A and B values as well as the respective p-value are presented for each microbial indicator. The analysis is based on microbial family level.

## Data Availability

Raw amplicon sequencing data have been deposited on NCBI’s SRA (sequence read archive; accession PRJNA716130). All other data generated or analyzed during the current study are included in the manuscript and its additional files. Reviewer Link: https://dataview.ncbi.nlm.nih.gov/object/PRJNA716130?reviewer=nddpiikt6t8ivk912j6kmi1nib

## Abbreviations

SCFA: Short chain fatty acids
ASV: Amplicon sequence variant
ENS: Effective number of species
CV: Coefficient of variation
